# Effects of Dufulin on Oxidative Stress and Metabolomic Profile of Tubifex

**DOI:** 10.3390/metabo11060381

**Published:** 2021-06-11

**Authors:** Yile Yu, Yuxin Zhu, Jing Yang, Wentao Zhu, Zhiqiang Zhou, Renke Zhang

**Affiliations:** Innovation Center of Pesticide Research, Department of Applied Chemistry, College of Science, China Agricultural University, Beijing 100193, China; 2018310060214@cau.edu.cn (Y.Y.); 2018310060209@cau.edu.cn (Y.Z.); 2018310060220@cau.edu.cn (J.Y.); wentaozhu@cau.edu.cn (W.Z.); zqzhou@cau.edu.cn (Z.Z.)

**Keywords:** metabolomics, urea cycle, argininosuccinate lyase, Tubifex

## Abstract

Dufulin is a highly effective antiviral pesticide used in plants. In this study, a seven-day experiment was conducted to evaluate the effects of Dufulin at five different concentrations (1 × 10^−4^, 1 × 10^−3^, 1 × 10^−2^, 0.1, and 1 mg/L) on Tubifex. LC-MS-based metabolome analysis detected a total of 5356 features in positive and 9110 features in negative, of which 41 showed significant changes and were identified as differential metabolites. Four metabolic pathways were selected for further study. Detailed analysis revealed that Dufulin exposure affected the urea cycle of Tubifex, probably via argininosuccinate lyase (ASL) inhibition. It also affected the fatty acid metabolism, leading to changes in the concentration of free fatty acids in Tubifex. Furthermore, the changes in metabolites after exposure to Dufulin at 1 × 10^−2^ mg/L were different from those at the other concentrations.

## 1. Introduction

Pesticides play an important role in human health protection and agricultural production [1]. Scientists have developed a variety of pesticides with higher effectiveness and lower toxicity in the past few decades [2]. Among the different pesticides, Dufulin ([(2-fluorophenyl)-(4-methylbenzothiazol-2-ylamino)-methyl]-phosphonic acid diethyl ester), an α-amino phosphonic acid ester [3], is a highly effective antiviral agent widely used against plant viruses. It was first developed in 2001 by Guizhou University [4,5]. Dufulin functions by activating the plants’ systemic acquired resistance (SAR) [3], aiming at Harpin binding protein-1 (HrBP1) [6]. Researchers have also found that Dufulin inhibits the RNA-silencing suppressor (P6) of the virus, especially the Southern rice black-streaked dwarf virus (SRBSDV) [7], protecting plants against the viral disease [8]. The Ministry of Agriculture of China has registered Dufulin as an antiviral pesticide for use in rice, tobacco, and vegetables [3]. Dufulin is an eco-friendly bioisostere of natural amino acids [3]. Pesticides sprayed on the field may stay on plants in small amounts, which eventually reach the soil [9,10]. Soil microorganisms degrade the residues of pesticides, including Dufulin [3]. However, the non-degraded residues pass from the soil into the water through runoff [11] or from crops to the air by volatilization and wind erosion and subsequently enter the rivers via rainfall [12]. These residues eventually reach the rivers [13] and deposit on the sediments [14,15]. Studies have identified approximately 65 different kinds of pesticides’ residues in the surface sediments of the China East Sea [16]. Therefore, the pesticide pollution of an aquatic environment should be urgently evaluated.

Sludge worm (*Tubifex tubifex*) is an aquatic oligochaete, which is ubiquitous in nature [17]. Due to the double-phase life mode, it plays an essential role in the aquatic ecosystem. They live in water and sediment [18]. The Tubifex ingests sediment particles and feeds on fine organic matter [19]. It can live in a wide variety of aquatic environments, ranging from pure undernourished water to hypereutrophic water [20], and can survive in a heavily polluted environment [21]. Higher organisms, such as fish, feed on Tubifex, and therefore, the sediment-related pollutants get transferred easily to higher trophic levels through the food chain [22,23]. Thus, Tubifex, an excellent bioindicator, is widely used in toxicologic research [24]. Several studies have used Tubifex to evaluate the toxicity of pesticides. Di. et al. [25] reported a positive effect of Tubifex on the degradation and diffusion of Metalaxyl in sediments. Li. et al. [26] found that Tubifex reduced the concentration of Dufulin in soil and accelerated the process of environmental remediation and detoxification. However, these studies have focused on the selective metabolism of pesticides in Tubifex. A more comprehensive assessment of the pesticide impact on non-target organisms, such as Tubifex, is necessary, and hence, the impact of pesticides on non-target organisms’ metabolism needs to be studied.

Metabolomics, an established omics [27], enables a comprehensive assessment. This method monitors the metabolic networks in vivo and detects small changes in the corresponding biomarkers [28,29]. It helps identify biomarkers and discover the driving forces of metabolic processes [30,31,32,33,34]. Metabolomics methodologies fall into two distinct groups: targeted metabolomics and untargeted metabolomics [35]. In targeted metabolomics, the relative concentration and richness of metabolites can be predefined quantitatively by monitoring the signals corresponding to preselected responses through various detection methods like tandem mass spectrometry (MS/MS) [35,36]. Targeted metabolomics provides an excellent quantitative analysis; however, the strategies adopted have limited coverage of metabolites [37,38]. Meanwhile, untargeted metabolomics focuses on the comprehensive detection of metabolites without bias [35,39]. Metabolomics, both targeted and untargeted, generally adopts techniques like nuclear magnetic resonance (NMR) [40] or mass spectrometry (MS) [41]. The original spectrogram is processed and analyzed to obtain the data, compared with a standard database (HMDB, KEGG) to obtain the results [41,42]. Currently, a combination of multiple platforms has been used for comprehensive analysis, such as the liquid chromatography-mass spectrometry (LC-MS)-based metabolomics [43,44]. The untargeted metabolomics uses LC-MS due to high-speed scanning and ideal analytic stability [38].

Oxidative stress, an important indicator of metabolic response and widely associated with metabolic changes, generates peroxides and free radicals and leads to cellular damage [45]. Recent studies have shown that harmful substances from outside, such as pesticides, stimulate the production of reactive oxygen species (ROS) in organisms [46]. These ROS interact with cell surface biomolecules [47], such as proteins and lipids [48], leading to loss or change in normal metabolic functioning [49]. Enzymes like SOD can eliminate these ROS and reduce oxidative damage [50]. However, oxidative damage disrupts the metabolic balance [51] and leads to changes in cell signaling. Therefore, the content of superoxide dismutase (SOD) and catalase (CAT) should be analyzed to determine the degree of oxidative stress damage [52] induced by xenobiotics, such as pesticides.

In this study, the toxicity of Dufulin on Tubifex was evaluated using the LC-MS-based metabolomics approach combined with oxidation damage detection. The study’s findings may help understand the mode of action of Dufulin and the mechanism of impact on Tubifex.

## 2. Results and Discussion

### 2.1. Effects of Dufulin on Oxidative Stress in Tubifex

**Figure 1:**
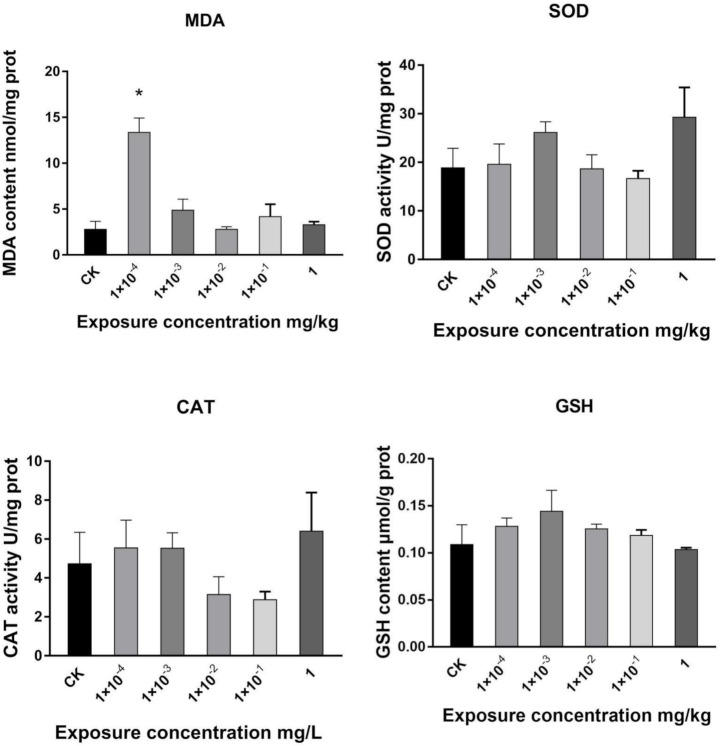
Changes in MDA, SOD, CAT, and GSH in Tubifex after exposure to Dufulin at different concentrations for 7 days. Each data set is represented as the mean value of three samples. Error bars indicate standard deviation (SD). (*: *p* < 0.05).

Pesticides induce oxidative stress by generating ROS [53]. Oxidative stress indicators, such as malondialdehyde (MDA), SOD, glutathione (GSH), and CAT, were used to evaluate the toxicological effect of Dufulin on Tubifex after seven days of exposure. Figure 1 shows that the MDA content increased significantly after Dufulin treatment at a concentration of 1 × 10^−4^ mg/L. No significant changes were observed in the other groups. Lipid peroxidation is an important indicator of oxidative damage in an organism [54]. The primary and secondary products of lipid peroxidation decompose and form MDA. Thus, MDA acts as an index of lipid peroxidation [55]. CAT is an antioxidant enzyme that eliminates excess hydrogen peroxide (H_2_O_2_) produced by oxidative damage [56]. Superoxide dismutase (SOD) protects the organism against oxidant damage, and therefore, SOD induction is associated with increased tolerance to oxidant stress [57]. Meanwhile, GSH, synthesized from glutamic acid, glycine, and cysteine [58], an abundant physiological nucleophile in cells [59], captures active heterometabolites through its cysteine sulfhydryl group and forms adducts under the action of glutathione transferases (GSTs) to protect cells [60]. However, in this study, no significant changes were observed in the GSH content and SOD and CAT activities. To conclude, the exposure of Dufulin at 1 × 10^−4^ mg/L increased MDA, with no significant change at other concentrations. This change may be related to lipid peroxidation. Whether Dufulin leads to the change of lipid peroxidation level still needs more data support. Meanwhile, SOD, CAT, and GSH showed no significant changes after exposure to Dufulin at any concentration. Under the exposure of Dufulin, the MDA content of Tubifex present a non-linear effect. The possibility of non-linear effect responses is in endpoints that are reflective of changes of metabolites rather than general toxicity [61].

### 2.2. LC-MS-Based Metabolomic Data

**Figure 2:**
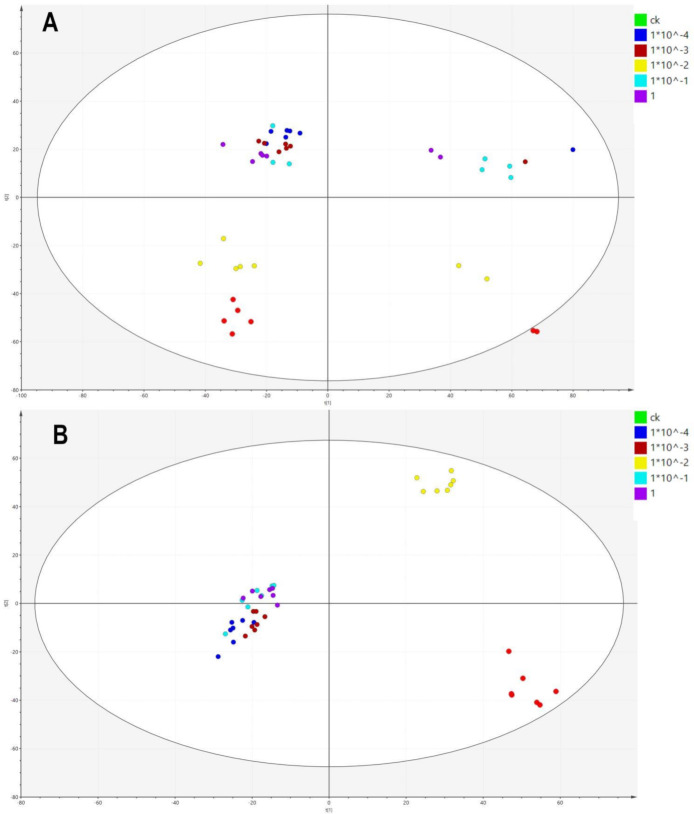
Statistical analysis of six groups (CK, 1 × 10^−4^, 1 × 10^−3^, 1 × 10^−2^, 1 × 10^−1^ mg/L). (**A**) Principal component analysis (PCA) score plot and (**B**) partial least-squares discriminant analysis (PLS-DA).

**Figure 3:**
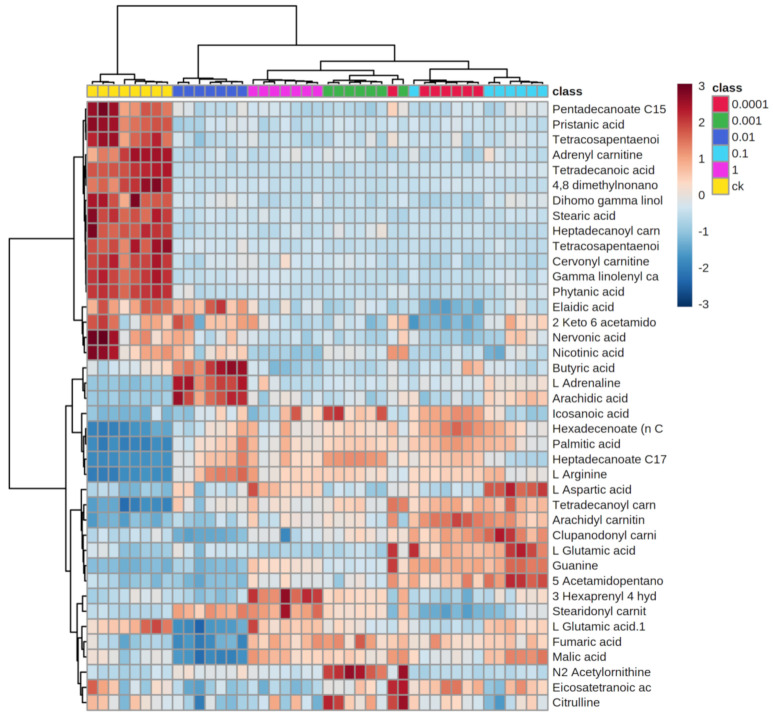
Heat map.

PCA was performed for an initial review of the data set and to detect outliers affecting the analysis. The analysis showed a clear separation of the control group (CK) and the different treatment groups (Figure 2A). Among them, the group exposed to 1 × 10^−2^ mg/L Dufulin was more obviously separated from the other groups. To further study the influence of Dufulin on Tubifex, a PLS-DA (Figure 2B) model was built to supervise the data analysis. The PLS-DA model was used to process the data, and the differences between treatment groups and control groups with various concentrations were analyzed. A heatmap (Figure 3) showed that the CK group and treatment groups were separated clearly.

### 2.3. Effects of Dufulin on Tubifex Metabolism

A total of 5356 features in positive and 9110 features in negative were obtained after processing the MS data using XCMS software (The Scripps Research Institute, La Jolla, CA, USA). Based on VIP (>1), *p*-value (<0.05), and fold change (>1.5), 41 differential metabolites were identified in each treatment group after exposure to Dufulin.

**Figure 4:**
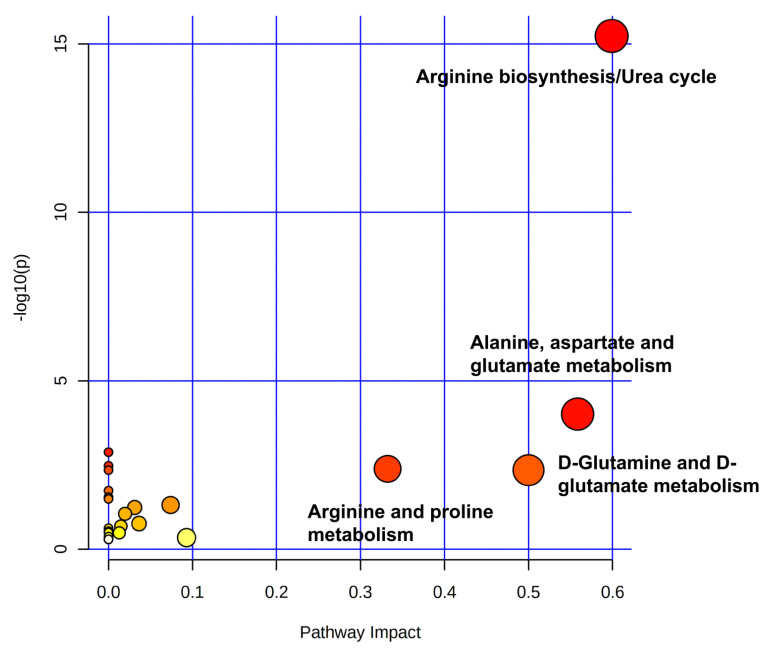
Metabolic pathway analysis by MetaboAnalyst. The pathway analysis was based on 41 unique metabolites. The *x*-axis represents the pathway impact value from pathway topology analysis, and the *y*-axis represents the -log *p*-value from pathway enrichment analysis. The node color and radius are based on *p*-value and pathway impact value, respectively.

MetaboAnalyst was used to perform the metabolic pathway analysis (Figure 4) using the 41 differential metabolites identified to understand the influence of Dufulin at different concentrations on Tubifex. Significant changes were observed in many compounds following Dufulin exposure.

Nervonic acid, pristanic acid, tetracosapentaenoic acid, tetradecanoic acid, palmitic acid, stearic acid, tetracosahexaenoic acid, phytanic acid, petadecanoate, and nicotinic acid levels decreased significantly after exposure to Dufulin, while heptadecanoate, hexadecenoate, and arachidic acid increased. The eicosatetraenoic content increased in groups exposed to Dufulin at 1 × 10^−4^ and 1 × 10^−1^ mg/L, while it decreased at other concentrations. At 1 × 10^−4^ and 1 × 10^−2^ mg/L of Dufulin, the butyric acid content increased (Figure S1), while it decreased at other concentrations. Nervonic acid is an essential functional long-chain fatty acid related to the neural system [62,63]. These changes of fatty acids in treatment groups reveal that the fatty acid activation pathway was affected after exposure to Dufulin at five concentrations used in this study.

**Figure 5:**
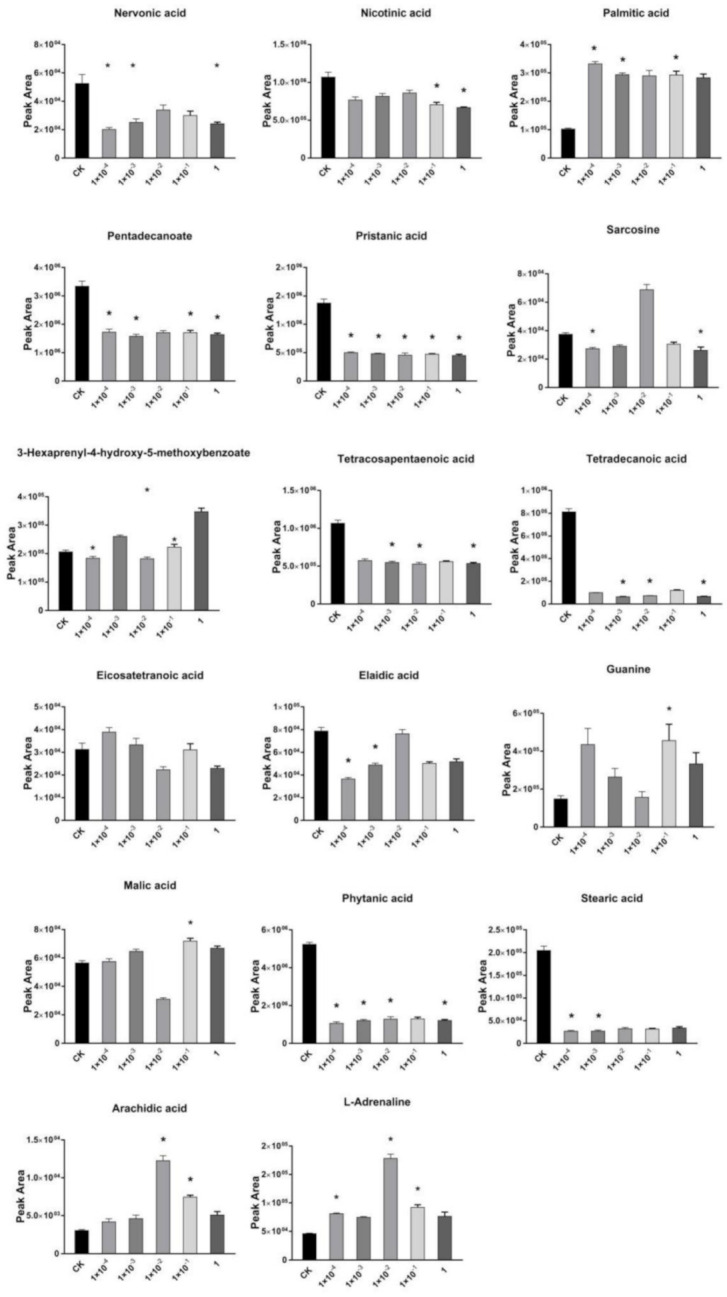
Changes in the concentration of metabolites of fatty acid in Tubifex exposed to Dufulin. Error bars indicate standard deviation (SD). (*: *p* < 0.05).

Activation of fatty acids is closely related to oxidative phosphorylation and plays an important role in fatty acid metabolism (Figure 5) [64]. The concentrations of arachidyl carnitine and tetradecanoyl carnitine were upregulated at the five concentrations (Figure S2). On the contrary, cervonyl carnitine, adrenyl carnitine, gamma-linolenyl carnitine, dihomo-gamma-linolenyl carnitine, and heptadecanoyl carnitine decreased. The content of tetracosatetraenoyl carnitine and tetracosapentaenoyl carnitine increased at 1 × 10^−2^ mg/L, while that at other concentrations decreased. However, the content of cupanodonyl carnitine and heptadecanoyl carnitine decreased at 1 × 10^−2^ mg/L Dufulin, while that at other concentrations increased. The content of stearidonyl carnitine decreased at 1 × 10^−4^ and 1 × 10^−1^ mg/L, while it increased at the other concentrations. The change of carnitine in the treatment groups reveals that the carnitine shuttle was affected upon Dufulin exposure. Carnitine transfers long-chain fatty acids into the mitochondria for β-oxidation [65]. Carnitine performs its scavenging function by binding to acyl residues derived from the intermediate metabolism of amino acids [66,67]. Carnitine is an important factor in regulating the cellular energy metabolism balance of fatty acids and glucose. In humans and other mammals, carnitine deficiency can lead to hyperlipidemia and systemic metabolic syndrome [68]. In this study, the exposure of Dufulin affected the transport of fatty acids, in which carnitine was involved, and affected the concentration of free fatty acids in Tubifex. This effect subsequently influenced Tubifex’s reaction to oxidative damage.

### 2.4. Impact of Dufulin on Tubifex Urea Cycle

**Figure 6:**
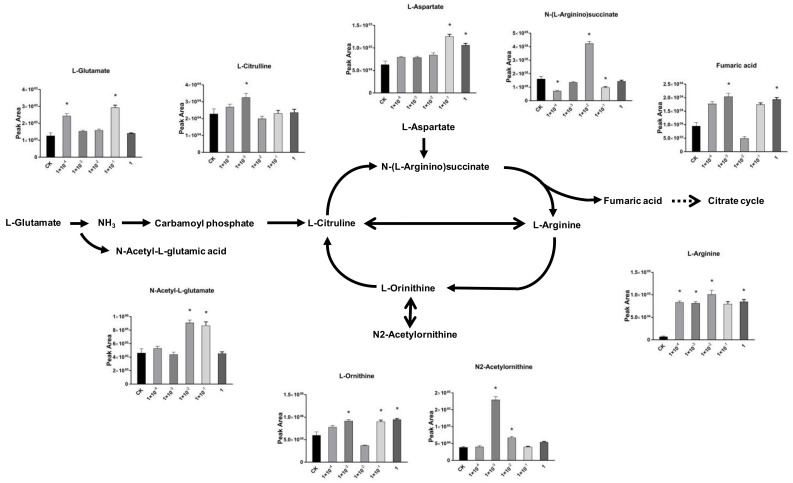
Schematic representation of the urea cycle exposed to Dufulin in Tubifex. Error bars indicate standard deviation (SD). (*: *p* < 0.05).

Among the different metabolic pathways in Tubifex, arginine biosynthesis was the most significantly affected at all concentrations. Nitrogen oxide (NO) is synthesized from arginine through a series of metabolic pathways [69]. Arginine plays several important metabolic roles, including the transport of nitrogen and the synthesis of protein via the urea cycle [70]. Arginine can be synthesized into ornithine under the action of arginase [71]. The urea cycle is a part of the amino acid metabolism in animals, and several pathways involving arginine metabolism overlap with the urea cycle [72]. To further explore the impact of Dufulin on Tubifex, the urea cycle, with more components affected by Dufulin, was analyzed (Figure 6). In the urea cycle, the metabolites showed different changing trends under different Dufulin concentrations. Metabolome analysis showed that the concentration of L-glutamate increased while that of downstream N-acetyl-L-glutamate decreased at 1 × 10^−3^ mg/L and 1 mg/L Dufulin exposure. Meanwhile, at 1 × 10^−2^ and 1 × 10^−1^ mg/L, N-acetyl-L-glutamate increased significantly. The content of L-citrulline increased at 1 mg/mL, while it dropped at the other concentrations. The content of L-ornithine decreased at 1 × 10^−2^ mg/L, while it increased at other concentrations. L-aspartate concentration increased in all treatment groups. The content of N-(L-Arginino)-succinate increased at 1 × 10^−2^ mg/L, while it decreased at other concentrations. The content of fumaric acid decreased at 1 × 10^−2^ mg/L and increased at other concentrations. The cleavage of N-(L-arginino)-succinate, catalyzed by the enzyme argininosuccinate lyase (ASL), is a key step in the urea cycle and arginine metabolism. Studies have shown that fluoride derivatives inhibit ASL. Fluoride derivatives form a tightly bound intermediate with ASL, which inactivates but does not kill the enzyme. The enzyme activity can be restored under appropriate conditions [73]. Thus, opposite trends were observed at 1 × 10^−2^ mg/L and other concentrations. ASL activity decreased at 1 × 10^−2^ mg/L, while it got activated at other concentrations. To conclude, Dufulin exposure had significant effects on the urea cycle of Tubifex. The increase in N-(L-arginino)-succinate and the decrease in fumarate revealed the inactivation of ASL. Thus, Dufulin probably acted on the urea cycle by inhibiting ASL, leading to urea cycle disorder. Earthworms with a complete urea cycle may be used to detoxify protein catabolism by increasing the production of excess ammonia [74]. In humans, the urea cycle disorder leads to the accumulation of ammonia and other neurotoxic byproducts that can lead to neurotoxicity and hyperaminemia [75], leading to irreversible neurological damage or death [76]. However, more research is needed to support the effect of urea cycle disorder in Tubifex.

## 3. Materials and Methods

### 3.1. Chemicals and Materials

Dufulin analytical standard (purity 98.5%) was obtained from the Institute for Guangxi Rural Research (Guangxi, China). Tubifex was purchased from the Beijing Da Senlin Flower Market (Beijing, China) [53].

### 3.2. Experimental Design

#### 3.2.1. Growth of the Experimental Organism

Tubifex worms were maintained in a 5 L beaker containing deionized water (temperature 21 ± 1 °C and no sediment, with continuous aeration provided using an aerator under laboratory conditions for 72 h for acclimatization before the experiment. The water was replaced daily.

#### 3.2.2. Exposure of Dufulin on Tubifex

The exposure experiment was conducted using six groups: CK and five treatment groups. One liter of water was added. In the treatment groups, Tubifex worms were exposed to Dufulin at 1 × 10^−4^, 1 × 10^−3^, 1 × 10^−2^, 1 × 10^−1^, and 1 mg/L concentrations. In the control group, the same volume of solvent was added. Three replicates were maintained per group. First, an average of 50 g (±10 g) of Tubifex was added to each beaker and exposed to natural light for 7 days at 18 °C (±1 °C). Water and pesticides were changed daily to maintain a constant exposure concentration. After seven days of exposure, the worms were removed from the beaker, and the dead remains were separated from the living individuals. The worms were frozen in liquid nitrogen, lyophilized, and stored at −10 °C.

#### 3.2.3. Sample Pretreatment for the Analysis of Oxidative Stress

Worms (1 g) were selected from each replicate of each treatment group and placed in a 2 mL centrifuge tube. Then, a stainless steel ball (5 mm diameter) was placed in the tube, and the worms were ground evenly using the Retsch MM 400 mixer grinder. To this grinding solution, 5 mL of 1 M phosphatic buffer solution (PBS; pH = 7.0) was added and vortexed for 30 s. The mixture was then centrifuged at 14,000 rpm for 20 min at 4 °C. The supernatant was separated, and the procedure was done twice to eliminate any particulate suspensions.

#### 3.2.4. Metabolomic Analysis of Tubifex by LC-Q-TOF

Freshly collected Tubifex worms were dried in a lyophilizer to constant weight, and the dried sample was placed in a 2 mL centrifuge tube. Then, a stainless steel ball (5 mm diameter) was placed in each tube, and the worms were ground evenly using a Retsch MM 400 mixer grinder. The homogenized tissue was mixed with 1.2 mL of 80% methanol, vortexed for 30 s, treated with ultrasound for 15 min, and placed at 4 °C for 30 min for thorough and even extraction. This mixture was centrifuged at 14,000 rpm and 4 °C for 20 min, and the supernatant was separated into a new tube with a pipette.

One milliliter of the supernatant was taken in a glass injection vial for metabolomic analysis. The metabolites were analyzed using the Agilent 1260 SL LC-6520 Quadrupole-Time of Flight (Q-TOF) MS system (Agilent Technologies, Santa Clara, CA, USA) [77,78,79,80].

A Waters XBridge BEH Amide column (15 cm × 2.1 mm, 2.5 μm) was used for separation. The mobile phase consisted of ACN and H_2_O, both containing 5 mM ammonium acetate and 0.1% acetic acid. Gradient elution was performed as follows: 95% ACN for 1.5 min, 95−78% ACN from 1.5 to 6.0 min, 78−50% ACN from 6.0 to 9.0 min, 50% B from 9.0 to 15.0 min, restoration to 95% ACN from 15.0 to 17.0 min, and continued 100% B from 17.0 to 30.0 to equilibrate the LC column.

The ESI conditions were as follows: electrospray ion-source ESI Agilent Jet Stream Technology in positive ionization mode; voltage 3.8 kV; desolvation temperature 325 °C; cone flow 20 L/h; desolvation gas flow 600 L/h; nebulizer pressure 45 psi, N2 drying gas; MS scan rate of 1.03 spectra/s across the range *m*/*z* 60–1000. Data were acquired using MassHunter Data Acquisition Workstation software (Agilent Technologies).

### 3.3. Biochemical Parameters Measurement

The MDA content, the catalase (CAT) and total superoxide dismutase (T-SOD) activities, the GSH content, and the total protein content of the tissue samples were measured using the kits purchased from Nanjing Jian Cheng Bioengineering Institute (Nanjing, China), following the manufacturer’s instructions. We measured the protein content of each Tubifex sample, so we divided each of measured content or activities by the total protein content. Three replicates were maintained per sample, and each sample was measured three times. One unit of SOD activity was defined as the quantity of enzyme required for 1 mg tissue proteins in 1 mL of a reaction mixture SOD inhibition rates to about 50% as monitored at 550 nm. One unit of CAT activities was defined as 1 mg tissue proteins consumed 1μmol H_2_O_2_ at 405 nm per second. The activities of SOD and CAT was demonstrated with U mg^−1^ proteins.

### 3.4. Data Analysis

The XCMS software (The Scripps Research Institute, La Jolla, CA, USA) was used for preliminary MS data processing, and ions with significant changes were extracted from the original data. Metaboananlyst 5.0 https://www.metaboanalyst.ca/, accessed on 5 June 2021), the online metabolomic analysis tool, was used to export data for multivariate statistical analysis. After deleting the unchanged peak, further analysis was carried out. The data of the obtained peak area was imported into SIMCA-P 13.0 (Umetrics, Umeå, Sweden) for multivariate statistical analysis. Pathway analysis was performed by the web-based MetPA tool (MetaboAnalyst Pathway Analysis; https://dev.metaboanalyst.ca/MetaboAnalyst/upload/PathUploadView.xhtml, accessed on 5 June 2021).

Principal component analysis (PCA) was performed to check for and rule out outliers. Furthermore, partial least square discriminant analysis (PLS-DA) was performed. The differential variables were characterized based on VIP (Variable Importance in Projection) scores calculated based on the PLS-DA model. Variables with VIP > 1 were the potential contributors to the grouping. The Kruskal-Wallis test was used to compare the means of the different treatment groups, and the differences were considered statistically significant at *p* < 0.05. The variables showing differences were ultimately determined at VIP > 1 and *p* < 0.05.

## 4. Conclusions

This study evaluated the effects of exposure of Dufulin at different concentrations on Tubifex by analyzing the oxidative damage and metabolome. Dufulin affected Tubifex at concentrations as low as 1 × 10^−4^ mg/L. It also affected the fatty acid metabolism, leading to changes in the concentration of free fatty acids. The urea cycle of Tubifex was also affected by Dufulin exposure while the possible mechanism via ASL inhibition. However, the effect of Dufulin on Tubifex was nonlinear. The study suggests that in untargeted organisms to pesticides, the impact of Dufulin even at low concentrations should be carefully considered. However, further studies should explore the mechanisms underlying the action of Dufulin at varying concentrations in Tubifex and verify whether the mechanism of the action of Dufulin is through the inhibition of ASL enzyme.

## Data Availability

The data presented in this study are available on request from the corresponding author. The data are not publicly available due to privacy.

## Supplementary Materials

The following are available online at https://www.mdpi.com/article/10.3390/metabo11060381/s1, Figure S1: Changes in the concentration of metabolites of butanoate metabolism in Tubifex exposed to Dufulin. Error bars indicate standard deviation (SD). (*: *p* < 0.05), Figure S2: Changes in the concentration of metabolites of carnitine shuttle in Tubifex exposed to Dufulin. Error bars indicate standard deviation (SD). (*: *p* < 0.05).
