# Supplementary material for: Effects of Dufulin on Oxidative Stress and Metabolomic Profile of Tubifex
Source: Metabolites. 2021 Jun 11;11(6):381. doi: 10.3390/metabo11060381 (PMC8231163; doi:10.3390/metabo11060381)
Supplement: Supplementary file 1 [file metabolites-11-00381-s001.zip › metabolites-1230332-supplementary.pdf]

Effects of Dufulin on Oxidative Stress and Metabolomic Profile of Tubifex

Yile Yu[1], Yuxin Zhu[1], Jing Yang[1], Wentao Zhu[1], Zhiqiang Zhou[1], Renke Zhang[1,*]

Supplementary Materials

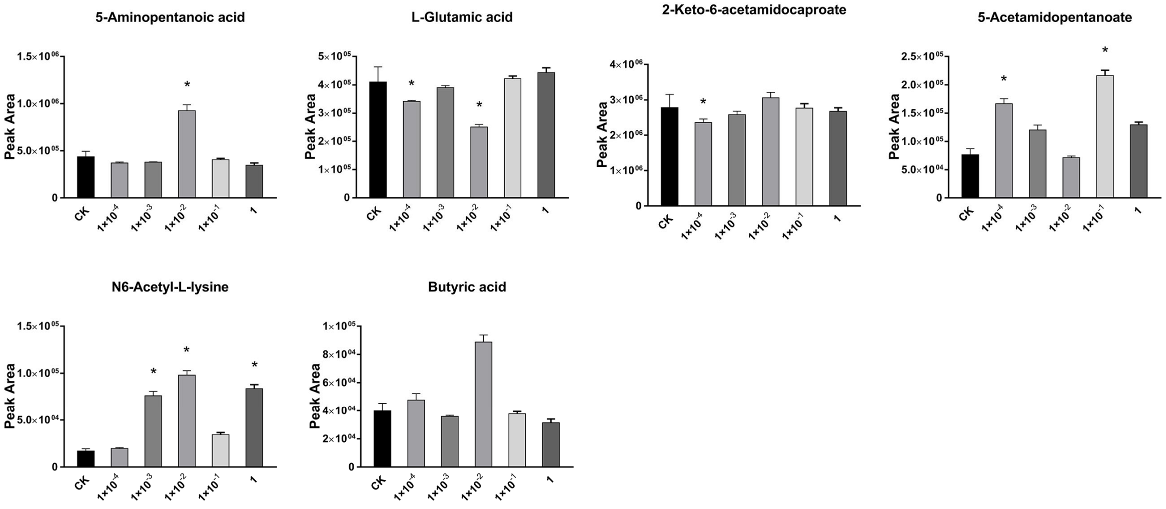

Figure S1. Changes in the concentration of metabolites of butanoate metabolism in Tubifex exposed to Dufulin. Error bars indicate standard deviation (SD). (*: $p < 0.05$).

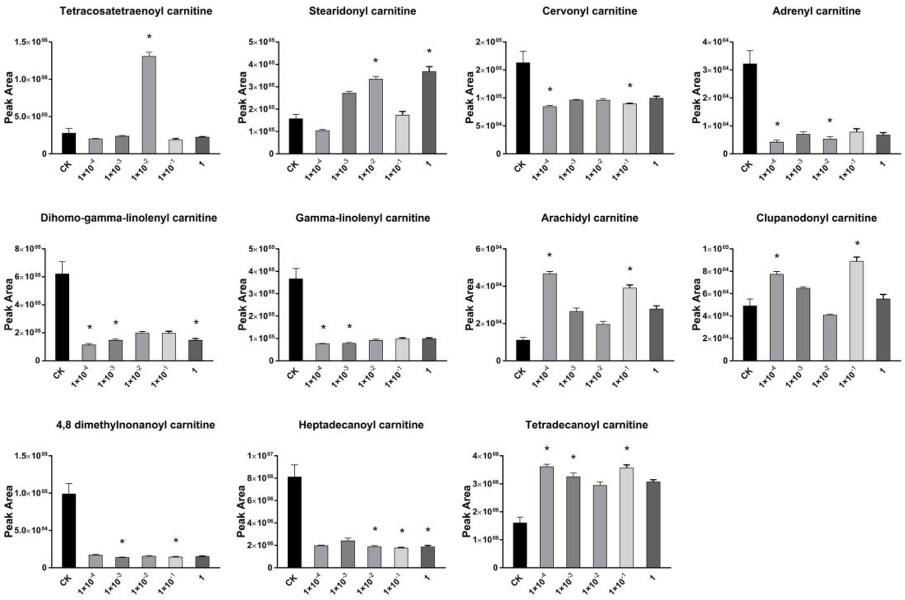

Figure S2. Changes in the concentration of metabolites of carnitine shuttle in Tubifex exposed to Dufulin. Error bars indicate standard deviation (SD). (*: $p < 0.05$).
